# “Violence. Enough already”: findings from a global participatory survey among women living with HIV

**DOI:** 10.7448/IAS.18.6.20285

**Published:** 2015-12-01

**Authors:** Luisa Orza, Susan Bewley, Cecilia Chung, E Tyler Crone, Hajjarah Nagadya, Marijo Vazquez, Alice Welbourn

**Affiliations:** 1 Salamander Trust, London, UK; 2 ATHENA Network, London, UK; 3 Women's Health Academic Centre, King's College London, London, UK; 4 Transgender Law Center, Oakland, CA, USA; 5 ATHENA Network, Seattle, WA, USA; 6 International Community of Women Living with HIV and AIDS (ICW) East Africa, Kampala, Uganda

**Keywords:** HIV, women, human rights, gender-based violence, values and preferences, survey, intimate partner violence, evidence base

## Abstract

**Introduction:**

Women living with HIV are vulnerable to gender-based violence (GBV) before and after diagnosis, in multiple settings. This study's aim was to explore how GBV is experienced by women living with HIV, how this affects women's sexual and reproductive health (SRH) and human rights (HR), and the implications for policymakers.

**Methods:**

A community-based, participatory, user-led, mixed-methods study was conducted, with women living with HIV from key affected populations. Simple descriptive frequencies were used for quantitative data. Thematic coding of open qualitative responses was performed and validated with key respondents.

**Results:**

In total, 945 women living with HIV from 94 countries participated in the study. Eighty-nine percent of 480 respondents to an optional section on GBV reported having experienced or feared violence, either before, since and/or because of their HIV diagnosis. GBV reporting was higher after HIV diagnosis (intimate partner, family/neighbours, community and health settings). Women described a complex and iterative relationship between GBV and HIV occurring throughout their lives, including breaches of confidentiality and lack of SRH choice in healthcare settings, forced/coerced treatments, HR abuses, moralistic and judgemental attitudes (including towards women from key populations), and fear of losing child custody. Respondents recommended healthcare practitioners and policymakers address stigma and discrimination, training, awareness-raising, and HR abuses in healthcare settings.

**Conclusions:**

Respondents reported increased GBV with partners and in families, communities and healthcare settings after their HIV diagnosis and across the life-cycle. Measures of GBV must be sought and monitored, particularly within healthcare settings that should be safe. Respondents offered policymakers a comprehensive range of recommendations to achieve their SRH and HR goals. Global guidance documents and policies are more likely to succeed for the end-users if lived experiences are used.

## Introduction

Evidence over the past 15 years, documented in peer-reviewed and informal literature, shows that women living with HIV globally experience high levels of gender-based violence (GBV) and human rights (HR) abuses, including structural violence [1–20]. Intimate partner violence (IPV), and violence against women, as well as stigma and discrimination, can be viewed as overlapping sub-sets of GBV [7,15]. There are varied definitions of GBV [7,15,21,22], with different combinations of physical, sexual, psychological, financial, legal or structural abuses. Only one, from a key 2011 document aiming to start documenting the forms and extent of violence they experience, relates specifically to women living with HIV: “violence against positive women is any act, structure or process in which power is exerted in such a way as to cause physical, sexual, psychological, financial or legal harm to women living with HIV” [15].

### GBV before HIV diagnosis

IPV is already recognized to increase during pregnancy for some women regardless of HIV status: this also increases risk of poor pregnancy outcomes [23]. The World Health Organization (WHO) and others [8,10] document how IPV can increase women's vulnerability to acquiring HIV. For example, forced sex, limited or compromised negotiation about safer sex and consequent increased sexual risk-taking can all result in women acquiring HIV [4]. Violence-related mental health problems can also result in HIV acquisition and progression [4].

### GBV after HIV diagnosis

Global HIV policies regarding women with HIV have remained largely silent on GBV experienced after HIV diagnosis [24–28]. Yet IPV can affect women's ability to initiate or adhere to treatment [20]. This may have policy implications on when best to start [27,29]. Moreover, other GBV consequences, including poor mental health, poverty and lack of widow's property and inheritance rights can particularly affect capacity for women to cope with HIV, with subsequent health consequences for their children also [7].

Healthcare is recognized as a setting where professionals can support women experiencing GBV [30]. However, HR abuses against women living with HIV in healthcare settings, such as forced and coerced sterilization [31] and related psychological violence, have been documented and represent manifestations of structural and GBV [17,18,21,22].

Balancing the impact of “formal” (scholarly/peer-reviewed/replicable) and “informal” (policy/activist/community-based) evidence is challenging, but there is ongoing value in ensuring the voices and experiences of women living with HIV are heard when developing policies which affect them. In 2014, the World Health Organization (WHO) commissioned a user-led and -designed global values and preferences survey of women living with HIV, to enable policymakers to update its 2006 Guidelines on the sexual and reproductive health (SRH) of women living with HIV [32]. These guidelines required updating through changing biomedical and political aspects of the HIV response, and internal WHO technical guidelines production processes [33]. WHO aimed to enhance the relevance and effectiveness of global guidelines, policies and programmes by exploring the lived experiences of women with HIV through involving and listening to women from the outset.

The aim of this article is to explore the ways women living with HIV experienced GBV. It describes how this impacted on their SRH and HR, as elicited from this community-based participatory research process, and implications of these finding for policymakers.

## Methods

### Overall survey development and dissemination

As community-based participatory research, this comprised an anonymous, confidential, mixed-methods, global, web-based values and preferences survey [34–36]. A Global Reference Group (GRG) of 14 women with diverse experiences of acquiring and living with HIV shaped the instrument using Survey Monkey, as described elsewhere [33,37]. The GRG used a written pre-survey exercise, developed by the core team, which reviewed women's lives over the lifecourse and to reflect on all facets (physical, psychological, material, sexual, spiritual) of women's lives in relation to their SRH. The results from the pre-survey exercise informed all the survey sections and questions. The survey used an appreciative inquiry approach (which focuses on the respondent as expert in creating positive, future-oriented outcomes) [38], grounded in HR [33] and sought to identify strategies which already work and can be expanded.

The resulting survey had one mandatory and eight optional sections, included quantitative and qualitative elements, was pilot-tested with iterative feedback by the GRG and disseminated in English, French, Spanish, Russian, Portuguese, Bahasa Indonesian and Chinese.

The process ran from February to June 2014, building on a non-random snowball sampling model developed by ATHENA Network [39], advertized and promoted through regional and global listservs of networks of women with HIV and clinical networks also [33]. The survey study population included any woman around the world living with HIV [37].

### Gender-based violence questions

The GRG identified GBV as a key issue. Questions on experiences of GBV (including within SRH services), gender inequality, harmful traditional/cultural practices, and violence from intimate partners/family members/community were thus included in the initial, mandatory section.

One optional section focused further on GBV [37]. This explored experiences and fear of GBV (from intimate partner, family and neighbours, wider community, health services, and prison/detention). Another question asked women to rank as “critical,” “important” or “less important” a series of policy and programme recommendations to address or prevent GBV that had been informed by the GRG regarding specific identities of women with HIV. Women were asked about their experiences of various types of GBV and when these were experienced in relation to HIV diagnosis (“before”, “since” and “because of” answer options were not mutually exclusive). Women were also offered freetext space to detail experiences of violence and how they had accessed support.

### Analysis

Simple descriptive frequencies and proportions were used for quantitative survey data. When answers were not mutually exclusive, “ever experienced” was calculated by subtraction of “never” and “don't know” from the total.

Freetext answers in all survey sections were translated back into English where necessary and reviewed for themes about experiences of GBV and for policy and programme recommendations [15]. Thematic coding of all open qualitative responses (survey freetext answers) was performed by one social scientist (LO), through manual textual analysis to identify emergent common themes, and validated with GRG members.

### Ethical considerations

Institutional review board approval for the survey was not sought, after discussion with the WHO Reproductive Health and Research Department and members of Guidelines Review Committee, as this study was a consultative element of the guidelines development process. Ethical considerations were undertaken, however, in line with the WHO 2001 *Ethical and Safety Recommendations for Research on Domestic Violence against Women* and the International Community of Women Living with HIV/AIDS 2004 guidelines on involving women with HIV in research [40,41].

Full explanations of the survey aims and purposes, including overviews of the structure and question areas, and definitions of SRH and HR, opened the survey. Respondents were required to confirm they had HIV and that they gave their consent for their responses to be used in these publications. Online respondents who did not click “I agree” could not proceed. These responses were not counted. Participation was taken as implicit consent.

## Results

### Survey participants

In total, 832 women living with HIV from 94 countries participated in an online survey [33]. Their ages ranged from 15 to 72 years, with most in their 30s (32%) and 40s (32%). Their diversity is shown in Table 1.

**Table 1:** Participant characteristics – online survey (*n*=832, 100%)

*Survey participants’ self-identification of diversity^a^*	
Heterosexual	524 (63%)
Inject/use or have injected/used drugs	116 (14%)
Do, or have done, sex work	116 (14%)
Lesbian or bisexual	46 (5.5%)
Transgender women	37 (4.5%)
Intersex	6 (0.7%)
Indigenous	67 (8%)
In stable relationships	374 (45%)
Have experienced any form of genital cutting or mutilation	42 (5%)
Have or have had active tuberculosis, malaria or hepatitis C (respectively)	108 (13%), 150 (18%), 137 (16.5%)
Have been incarcerated or detained	42 (5%)
Have migrated for economic or political reasons (respectively)	158 (19%), 21 (2.5%)
Have other disabilities	100 (12%)
Are or have been homeless	116 (14%)

a Numbers do not add up to 100% as these were optional questions.

### Quantitative responses

GBV was reported in every section of the survey [33]. In the one mandatory section, participants overwhelmingly agreed that addressing GBV in all three contexts (within SRH services; in the home and community; and harmful traditional practices) was an “absolute must” or “high priority” (555–559/589: 94–97%). Few (18–34/589: 3–6%) considered addressing GBV to be “lower priority.”

The optional GBV section was completed by over half the online survey respondents (480/832, 58%). Table 2 shows the types, frequencies and timings of violence reported. For all six categories, some respondents gave multiple answers according to when the violence had occurred. Of respondents to the optional section, 89% (422/480) had experienced at least one form of violence in any of the six categories. Only 58 respondents (11%) said they had “never” experienced any form of violence (six of these ticked “never” in all six areas and at least one other response, but were counted as not having experienced violence). 100% (57) of respondents answering this section from Eastern Europe and Central Asia experienced at least one form of violence.

**Table 2:** Categories, frequencies and timings of violence reported by women with HIV

Category of violence experienced^a^	Before HIV diagnosis *n* (%)	Since HIV diagnosis *n* (%)	Because of HIV diagnosis *n* (%)	Any experience *n* (%)	Never *n* (%)	Don't know *n* (%)	Total *n* (%)
From a sexual partner or spouse	208 (43)	80 (17)	70 (15)	282 (59)	181 (38)	17 (4)	480 (100)
From a family member/neighbours	75 (16)	80 (17)	112 (24)	215 (45)	244 (51)	16 (3)	475 (100)
In the community	76 (16)	109 (23)	146 (32)	250 (53)	196 (42)	25 (5)	471 (100)
In health settings	28 (6)	133 (28)	164 (35)	253 (53)	209 (44)	13 (3)	475 (100)
From police/military/prison or detention services	44 (9)	34 (7)	26 (6)	78 (17)	360 (77)	31 (7)	469 (100)
Fear of violence	118 (25)	136 (29)	184 (39)	322 (68)	140 (30)	11 (2)	473 (100)

a Categories in columns 2 to 4 are not mutually exclusive. Only columns 5 to 7 add up to 100% across each row.

Fear of violence was the most commonly reported form of GBV (322/473, 68.1%), but only 15 (3%) reported “fear of violence” alone. The least reported form of GBV was from police/military/prison or detention (16.6%) (Table 2). The commonest form of violence before diagnosis was IPV (43%), which was exacerbated afterwards for some. Reports of four types of GBV (from family/neighbours, community, health settings, and fear of violence), were higher “since” or “because of” HIV diagnosis than “before.”

### Qualitative responses

In the one mandatory survey section, violence emerged as a key theme in freetext responses to the question “What's the most important issue that you would like to see the WHO Guidelines address in order to make it the most useful tool for you?” Answers varied from one word, “violence” (multiple responses), to longer statements concerning ways GBV intersects with HIV:

The impact of violence and the trauma associated with being a girl/woman in many parts of the world affect every aspect of our fight against HIV – from risk to diagnosis to accessing care to managing treatment to survival. (US)

### Lifelong violence

The optional GBV survey section also revealed that violence is not a one-off occurrence, and that it cannot be easily packaged as either a cause or consequence of HIV. The freetext results indicate the wide breadth of forms of GBV and its complex and iterative relationship with HIV, occurring throughout women's lives:

Before HIV, I was victim of different types of violence (physical, psychological, financial) besides the impact my partner's alcoholism and machismo; this lead me to get several STIs, including HIV. (El Salvador)

Violence occurred both before and after my diagnosis and post diagnosis the violence was related to stigma, which in turn resulted in being discriminated against, gossiped about, treated badly by family, asked to not use utensils, etc …. (Canada)

When I was newly diagnosed and had lost about 40Kg my neighbours and members of my church choir started avoiding me and in fact disallowed their children to come to my home and my son to enter theirs. It was such a painful experience for me. (Nigeria)

### Violence within healthcare settings

HIV diagnosis and disclosure can act as specific triggers for violence. They also expose women to new settings of HIV-related violence, including within healthcare institutions, especially concerning SRH. In other survey sections, women also reported confidentiality breaches leading to involuntary disclosure (exposing women to further forms of violence from partners, family and community members): “No Confidentiality. Nurse at hospital told my whole family. Lost my family business” (Belize/US).

Also from freetext responses, women indicated that their choice whether to test for HIV, and whether, when and how to disclose their status to their partner(s) was often compromised in maternity contexts:

Service providers should stop forcing women with HIV to disclose to their husbands, they should teach on how to help them to disclose, cause if a woman doesn't disclose she is not attended to. (Uganda)

### Lack of SRH choice

Whilst many respondents reported improved SRH services over the last 20 years, many still experienced negative provider attitudes regarding fertility desires, and no access to a full choice of services relating to contraception, maternity care and obstetrics, fertility treatment and adoption. Freetext survey responses included: “After my delivery, the midwives, knowing that I was HIV positive, didn't direct me towards family planning services … according to them I no longer had the right to have children” (Cote D'Ivoire).

The moment a woman identifies herself as living positively with HIV, they are neglected especially during delivery hence increased number of children born with HIV because women prefer to keep it a secret and be treated like the rest. Others have avoided giving birth from health centers … because of negligence in those hospitals. They prefer traditional birth attendants. (Uganda)

Respondents also reported mixed experiences regarding contraceptive choice. Service providers reportedly told many only to use condoms. Others have been coerced or forced into using long-acting or permanent birth-control methods, including intra-uterine devices (IUD, coils), injectable hormonal contraceptives, or tubal ligation (sterilization). Once diagnosed, women also reported treatment refusal (especially fertility treatment) or being forced or coerced into services they did not freely choose, including abortion: “When I got pregnant with my daughter, doctors tried to convince me to abort” (Portugal).

I went to the health facility for an IUD which they did willingly but after 6 months I wanted it removed because I wanted to have a baby. They told me the family planning method I used was for 12 yrs so they gave me a hard time that I wasted their IUD which another person would have used, ‘someone who is not like me’. It hurt me so much and I left. So I gave up removing it. (Uganda)

Make sure that there is a law that can heavily penalize doctors who perform forced sterilization and more; in many cases like mine we only realize many years later and [then] you cannot do anything. (Mexico)

### Human rights abuses and diversity

HR abuses were documented throughout the survey. In the human rights section, 180/589 (30%) respondents disagreed or strongly disagreed with the statement: “I experience the same service as any other women when I go for sexual and reproductive health services.” Their freetext responses included: “When it comes to referral to reproductive sexual health, STIs etc., the staff is completely uninformed and discrimination still persists, leaving care of women living with HIV til last” (Peru).

Women often faced considerable ignorance from health providers regarding SRH rights, and judgemental attitudes. This was exacerbated for sex workers, lesbians and transgender women especially around fertility and family planning access:

Because I am a sex worker I am ignored most of the times. (Malawi)

Health providers have no experience in dealing with lesbian women especially those living with HIV. … When asked to come with my “male partner” I responded that I am lesbian. The [healthcare professional] was very much disgusted when I revealed my sexual preference. I have never attended that hospital again. (Kenya)

As a trans woman, healthcare providers do not have the correct information about my body. They are very uncomfortable dealing with a woman who has a penis. … Now I avoid such centres. (South Africa)

Being Trans and HIV+ makes getting compassionate and comprehensive care very difficult. I have been hospitalized at the University of X Hospital, Y University Hospital, and University of Z Hospital – all places made me feel extremely unsafe. (US)

Women who used drugs also reported considerable stigma, discrimination and violence, especially regarding their reproductive rights. These respondents reported high anxiety, particularly over child custody by free text: “When my mother-in-law found out about my HIV-positive status from the hospital nurses, she made every effort to take away my daughter” (Russia/Ukraine).

When I was diagnosed doctors actively discouraged me from having a baby – even if we already knew good interventions to stop vertical transmission. Maybe it was because I was an ex-drug user and also had Hepatitis C Virus … But it took me many years to even ask again if I could have children & it was too late. (Italy)

### Support for policies to address or prevent GBV

Survey respondents were asked what messages to offer policymakers, or to give recommendations on how to address and prevent violence against women with HIV:

I would like them to address gender violence because women are suffering in the hands of their husbands. Most women like me stay in the marriage not because I want but because I have nowhere else to go and also the business I do is family business so if I leave I won't have any financial support, so I endure the beatings, insults etc. because I don't have an alternative. (Kenya)

### Policy recommendations

Women's responses to the policy recommendations presented in the optional GBV section covered a wide range of issues (Table 3). Over 80% of respondents agreed that these are critical or important ways healthcare services can address violence against women with HIV [33].

**Table 3:** What women living with HIV think are the most important ways to address or prevent gender-based violence

Strategy	Critical *n* (%)	Important *n* (%)	Less important *n* (%)	Don't know *n* (%)	Total response *n* (%)
*Through safe health services that protect, respect and uphold women's rights*					
Sensitize healthcare workers to the rights of women living with HIV	363 (77)	86 (18)	11 (2)	11 (2)	471 (100)
Increase access to quality support services for women who experience gender-based violence (including sexual violence)	356 (76)	94 (20)	11 (2)	8 (2)	469 (100)
Ensure effective complaints/redress mechanisms in case of rights violations within health services	332 (71)	114 (24)	10 (2)	10 (2)	466 (100)
Provide a minimum post-rape care and support package, including post-exposure prophylaxis, emergency contraception, screening for other sexually transmitted infections, and psychosocial care/counselling	330 (71)	114 (24)	9 (2)	13 (3)	466 (100)
Increase access to harm reduction-based treatment for women who use drugs	238 (51)	178 (38)	26 (6)	23 (5)	465 (100)
Address alcohol abuse	206 (44)	182 (39)	54 (12)	22 (5)	464 (100)
*Through a protective legal and policy environment and decriminalization*					
Strengthen laws and policies to protect the rights of people living with HIV	376 (80)	79 (17)	9 (2)	8 (2)	472 (100)
Strengthen legal protections around all forms of violence against women/gender-based violence	358 (76)	90 (19)	9 (2)	14 (3)	471 (100)
Recognize and address marital rape and “date rape”	280 (60)	143 (31)	17 (4)	25 (5)	465 (100)
Remove laws which criminalize HIV exposure/transmission	235 (51)	112 (24)	63 (14)	48 (10)	458 (100)
Remove laws which criminalize same sex practices	181 (40)	131 (29)	97 (21)	48 (11)	457 (100)
Remove laws which criminalize sex work	166 (35)	153 (33)	89 (19)	61 (13)	469 (100)
Remove laws which criminalize drug use	157 (34)	148 (32)	106 (23)	54 (12)	465 (100)
*Through financial security*					
Increase social protection for women and children	333 (71)	116 (25)	12 (3)	8 (2)	469 (100)
Increase access to employment for women, including transgender women	269 (58)	153 (33)	26 (6)	17 (4)	465 (100)

In looking beyond “just” an end to GBV, further policy and practice suggestions were ranked as shown in Table 4. A range of 54–93% of respondents agreed proposed measures were “critical” or “important.” For lower percentage responses, a consistently greater proportion also answered “don't know” (up to 30%).

**Table 4:** Policy recommendations that would improve the SRH of women living with HIV in all their diversity (from optional section relating to diversity)

Policy recommendations that would improve the SRH of women living with HIV in all their diversity	Proportion endorsing recommendations as critical or important *n* (%)
*Women who use drugs*	
Access to methadone or buprenorphine for women living with HIV who inject drugs and are pregnant	250/430 (56)
Treatment and support for hepatitis C co-morbidities	398/428 (93)
Education on prevention and first-aid for overdoses, including access to naloxone	*
*Sex workers*	
Interventions to halt and address violence and discrimination against sex workers	375/425 (88)
*Lesbian, bisexual, transgender women, and other women who have sex with women*	
SRH services tailored for lesbian, bisexual, transgender women or other women living with HIV who have sex with women	288/428 (67)
Introduction of SRH guidelines/policy for transgender women	279/419 (67)
Access to sexual reassignment surgery for transgender women	234/424 (55)
Access to other gender-affirming surgeries for transgender women	226/422 (54)
*Women in prison or detention*	
Continuity of treatment access and adherence support for women in prison or detention and women re-entering into society	393/426 (92)
Addressing HIV-related stigma and discrimination among prison staff and inmates	399/428 (93)
Consistent implementation of up-to-date practice guidelines in relation to women living with HIV in prison	388/426 (91)
*Women with disabilities*	
Tailored access to information and services for women with disabilities	402/431 (93)
*For all women with HIV, throughout the life-cycle*	
Comprehensive sexuality education	403/433 (93)
Removal of age-restrictive polices	254/403 (63)
Treatment and support for TB co-morbidities	398/428 (93)

* Inadvertently not included in the survey, but later highlighted by GRG members.

One survey respondent highlighted the importance of using the guidelines in conflict-afflicted countries:

Issues around GBV and HIV/AIDS for women and girls in South Sudan. The … country continues to be troubled in political issues and insecurity, women and girls continue to suffer the brunt of it all. WHO should be specific … on how to address GBV and HIV/AIDS for women and girls in an insecure country. (South Sudan)

## Discussion

This mixed-methods study, developed and led by women living with HIV, describes the experiences of 945 participants from 94 countries globally. Eight-hundred and thirty-two women took part in an online survey, the data from which are described above. A further 113 women living with HIV from key affected populations took part in 11 focus group discussions (FGDs) in seven countries. This enabled more respondents with no limited literacy or no computer access or limited literacy to answer the same survey questions and, through discussion, to deepen understanding and triangulate findings. Data analysis did not produce significant differences in responses between online survey respondents and FGD participants, therefore supporting the content validity of the online survey findings (data not shown). It is the largest study of its kind to explore SRH and HRs of women with HIV.

Of concern is that 89% of respondents to the GBV section of the online survey reported violence. Women reported HIV-related violence before, since and because of HIV diagnosis – or a combination of all three. Fear of violence was more common than other forms of GBV, but there were high background IPV levels before and after diagnosis. Notably, higher levels of violence were experienced post-diagnosis in health settings and the community. Peer-review literature to date has not highlighted the increased levels of GBV in such settings, nor the very high proportion of respondents reporting violence at different stages of their lives. GBV was a high priority for many women with HIV, including sex workers, transgender women and women who used drugs. The range and complexity of the violence experienced by women living with HIV combined to constrain their capacity to enjoy SHR and HRs.

The strengths of this largest-ever survey of women with HIV include being user-led, with efforts made to ensure inclusion of women living with HIV in multiple contexts and diversities. It was based on a snowballing technique via informed networks, used both qualitative and quantitative methods, and achieved a large global response.

With constrained timeframe and budget, the study's key limitation is that participants do not represent all women with HIV and was biased towards women with Internet access who had an activist interest through their listserv membership. Experience of GBV might influence the wish to participate in either direction. Numbers from each of many key populations are relatively small due to the breadth of participation. It was particularly difficult to reach younger women and women living with HIV in conflict-affected situations, who may have other specific experiences of violence not captured by the survey. Nevertheless, the response exceeded expectations and achieved sufficient breadth and depth of information required to support generalizability.

The implications for clinicians and policymakers are: that stigma and discrimination against women living with HIV are forms of GBV and need to be recognized as such; and that GBV is commonly experienced by women living with HIV globally. Safety is the foundation of an effective response to violence against women in the context of HIV. WHO/UNAIDS have documented the existing evidence base for effective interventions in four strategic areas: effective laws and policies, transformative gender norms, integrated violence against women and HIV services, and empowering women and girls though integrated multi-sectoral approaches [7]. Survey respondents highlighted all these strategic areas, and also others including alcohol policies and peer-support.

Healthcare settings need to be safe places for all women including key populations [42], and there is a need for greater awareness-raising and evidence-informed debate in these areas.

Healthcare practitioners and policymakers should be trained to explore the effects of their words and actions, both on the women themselves and on their ability to care for their children. Moreover, policies related to antiretroviral initiation must consider, measure and mitigate the GBV that may follow an HIV diagnosis.

Since so many women are diagnosed with HIV during pregnancy, healthcare practitioners and policymakers need to be particularly mindful of the incremental effects of a diagnosis on GBV. Considering the reports of lack of safety and HR protection within healthcare services, HR violations including mandatory testing, disclosure, and coerced or forced sterilization and abortion should be particularly urgently addressed. Such information might be sought directly (e.g. via community surveys, non-governmental organizations, legal redress, complaints), or found indirectly (e.g. from measuring access to care and treatment, non-attendance or subsequent retention in care) [43].

Implications also include that women living with HIV need to be aware of their rights and the different forms of GBV, and that they may identify and name violence, recognize rights violations, find support and develop greater agency to seek redress and safety. In addition to the priorities identified in the survey, this study supports suggestions that all healthcare staff should also be equipped to enquire about GBV, as a part of routine, everyday, safe interactions within healthcare settings [44,45], as benefit for all women and girls, especially those with HIV. Healthcare services must also have formal pathways to advocacy and safety planning for women who disclose GBV [44,45].

Regarding future research, there is urgent need for replication, quantification, refinement and testing of interventions informed by the experiences of violence and the intersections of health and HR across the life-cycle. Research should address health, dignity, and welfare; the interface between violence and HIV exposure, acquisition and impact; the interface between violence and other factors, such as gender identity and gender expression; their impact on the SRH and HR of women living with HIV; women's capacity to take antiretrovirals – as well as their ability to act as primary carers of the next generation.

## Conclusions

Women living with HIV experience unacceptable levels of violence, including in healthcare settings, across their life-cycle. This study contributes to the growing literature on GBV in women living with HIV, both before and after diagnosis. Global policymakers should heed this literature in policy formulation and implementation. Since guidelines examine the formal evidence base which takes time to be published, any time-lag in information or implementation risks failing the very end-users that global polices seek to support in the absence of such key information. Community-based information can act as a valuable early-warning monitoring system to assess unintended consequences of global policies.

No woman, with or without HIV, should have to experience GBV, especially not in healthcare settings, over which policymakers and service providers have most control. GBV should be recognized as a barrier to effectiveness of many policies regarding testing, care, treatment initiation and adherence. Addressing GBV as a critical policy issue has instrumental as well as inherent value. Forthcoming guidelines should encompass recommendations for policy, formal healthcare, and a more holistic view of the current SRH and HR of women living with HIV in all their diversities, which include community engagement, a firm foundation of safety, and are based on a HR framework.

## References

[1] Siemieniuk R, Krentz H, Miller P, Woodman K, Ko K, Gill M (1999). The clinical implications of high rates of intimate partner violence against HIV-positive women. J Acquir Immune Defic Syndr.

[2] Gielen A, McDonnell K, Burke J, O'Campo P (2000). Women's lives after an HIV-positive diagnosis: disclosure and violence. Matern Child Health J.

[3] Zierler S, Cunningham WE, Andersen R, Shapiro MF, Nakazono T, Morton S (2000). Violence victimization after HIV infection in a US probability sample of adult patients in primary care. Am J Public Health.

[4] Campbell JC, Baty ML, Ghandour RM, Stockman JK, Francisco L, Wagman J (2008). The intersection of intimate partner violence against women and HIV/AIDS: a review. Int J Inj Contr Saf Promot.

[5] Osinde M, Kaye D, Kakaire O (2011). Intimate partner violence among women with HIV infection in rural Uganda: critical implications for policy and practice. BMC Womens Health.

[6] Dhairyawan R, Tariq S, Scourse R, Coyne K (2012). Intimate partner violence in women living with HIV attending an inner city clinic in the UK: prevalence and associated factors. HIV Med.

[7] WHO/UNAIDS (2013). 16 ideas for addressing violence against women in the context of the HIV epidemic: a programming tool [Internet].

[8] (2013). Global and regional estimates of violence against women: prevalence and health effects of intimate partner violence and non-partner sexual violence [Internet].

[9] Li Y, Marshall C, Rees H, Nunez A, Ezeanolue E, Ehiri J (2014). Intimate partner violence and HIV infection among women: a systematic review and meta-analysis. J Int AIDS Soc.

[10] Durevall D, Lindskog A (2015). Intimate partner violence and HIV in ten sub-Saharan African countries: what do the Demographic and Health Surveys tell us?. Lancet Global Health.

[11] Zunner B, Dworkin S, Neylan T, Bukusi E, Oyaro P, Cohen C (2015). HIV, violence and women: unmet mental health care needs. J Affect Disord.

[12] Olowookere S, Fawole O, Adekanle D, Adeleke N, Abioye-Kuteyi A (2015). Patterns and correlates of intimate partner violence to women living with HIV/AIDS in Osogbo, Southwest Nigeria. Violence Against Women.

[13] Feldman R, Manchester J, Maposhere C, Cornwall A, Welbourn A (2002). Positive women: voices and choices in Zimbabwe. Realizing rights.

[14] (2007). Hidden in the mealie meal: gender-based abuses and women's HIV treatment in Zambia [Internet]. Human Rights Watch.

[15] Hale F, Vazquez M (2011). Violence against women living with HIV/AIDS: a background paper [Internet].

[16] Vazquez M, Hale F (2011). Ethical considerations for an integral response to human rights, HIV and violence against women in Central America [Internet].

[17] (2012). If I knew what would happen I would have kept it to myself: gender violence and HIV [Internet].

[18] Paxton S (2012). Positive and pregnant: how dare you – a study on access to reproductive and maternal health care for women living with HIV in Asia [Internet].

[19] Hutchinson J, Perry G (2012). Violence as a cause and consequence of women living with HIV in England [Internet].

[20] Mwanza J (2012). Baseline report on intimate partner violence amongst people living with HIV [Internet].

[21] Galtung J (1969). Violence, peace, and peace research. J Peace Res.

[22] Farmer PE, Nizeye B, Stulac S, Keshavjee S (2006). Structural violence and clinical medicine. PLoS Med.

[23] (2011). Intimate partner violence during pregnancy: information sheet [Internet].

[24] (2011). Global plan towards the elimination of new HIV infections among children by 2015 and keeping their mothers alive 2011–2015 [Internet].

[25] Chitembo A, Dilmitis S, Edwards O, Foote C, Griffiths L, Moroz S (2012). Towards an HIV-free generation: getting to zero or getting to rights?. Reprod Health Matters J.

[26] (2013). Consolidated guidelines on the use of anti-retroviral drugs for treating and preventing HIV infection: recommendations for a public health approach [Internet].

[27] Welbourn A (2014). WHO's poor consultation with patients on HIV guidance has denied women choice in drug treatment. BMJ.

[28] (2015). Guideline on when to start antiretroviral therapy and on pre-exposure prophylaxis for HIV [Internet].

[29] AVAC, ATHENA, Salamander Trust, UNWomen (2015). Key barriers to women's access to HIV treatment: making ‘Fast-Track’ a reality [Internet].

[30] Heise L, Ellsberg M, Gottmoeller M (2002). A global overview of gender-based violence. Int J Gynecol Obstet.

[31] Southern Africa Litigation Centre (2013). African Commission on human and peoples’ rights condemns involuntary sterilisation of women living with HIV in Africa [Internet].

[32] (2006). Sexual and reproductive health of women living with HIV/AIDS: guidelines on care, treatment and support for women living with HIV and their children in resource-constrained settings [Internet].

[33] Orza L, Welbourn A, Bewley S, Crone E, Vazquez M (2015). Building a safe house on firm ground: key findings from a global values and preferences survey regarding the sexual and reproductive health and human rights of women living with HIV [Internet].

[34] Raab M, Stuppert W (2014). Review of evaluation approaches and methods for interventions related to violence against women and girls (VAWG).

[35] Creswell J, Klassen A, Clark V, Smith K NIH Office of Behavioral and Social Sciences Research (OBSSR) – mixed methods research index [Internet].

[36] Jagosh J, Bush P, Salsberg J, Macaulay A, Greenhalgh T, Wong G (2015). A realist evaluation of community-based participatory research: partnership synergy, trust building and related ripple effects. BMC Public Health.

[37] (2014). Salamander Trust Values and Preferences Survey (Questionnaire): sexual and reproductive health and human rights for women living with HIV [Internet].

[38] McAdam E, Lang P (2009). Appreciative work in schools.

[39] (2011). In women's words: HIV priorities for positive change [Internet].

[40] (2001). Putting women first: ethical and safety recommendations for research on domestic violence against women [Internet].

[41] (2004). Guidelines on ethical participatory research with HIV positive women [Internet].

[42] (2014). Consolidated guidelines on HIV prevention, diagnosis, treatment and care for key populations [Internet].

[43] Whiteford L, Vindrola-Padros C (2015). Community participatory involvement.

[44] (2013). Responding to intimate partner violence and sexual violence against women WHO clinical and policy guidelines [Internet].

[45] (2014). Domestic violence and abuse: how health services, social care and the organisations they work with can respond effectively [Internet].

